# Engineered levothyroxine dry powder for inhalation to treat idiopathic pulmonary fibrosis

**DOI:** 10.1007/s13346-026-02064-2

**Published:** 2026-02-07

**Authors:** Gianluca Bazzoli, Annalisa Bianchera, Giovanna Trevisi, Elias Fattal, Juliette Vergnaud, Ruggero Bettini

**Affiliations:** 1 Food and Drug Department, University of Parma, Parco Area Delle Scienze 27/a, Parma, Italy; 2 Interdepartmental Center Biopharmanet-Tec, University of Parma, Parco Area Delle Scienze Building 33, Parma, Italy; 3 IMEM-CNR, Parco Area Delle Scienze 37/a, Parma, Italy; 4 Université Paris-Saclay, CNRS, Institut Galien Paris-Saclay, 91400 Orsay, France

**Keywords:** Idiopathic pulmonary fibrosis, Local lung delivery, Thyroid hormones, Nanoparticle, Dry powders for inhalation

## Abstract

Interest in drug repurposing has increased significantly in recent decades owing to its potential to accelerate the development of new medicinal products, provide new therapeutic options for patients, and generate business opportunities for pharmaceutical companies. Idiopathic pulmonary fibrosis (IPF) is defined as a chronic disease that causes an irreversible loss of lung function and premature death. Recent studies have highlighted the key role of mitochondria in lung homeostasis and the ability of thyroid hormones to promote mitochondrial activity, suggesting their potential involvment in IPF pathogenesis. In this work, we translate the findings derived from the above-mentioned researches into a dry powder drug delivery system intended to target epithelial lung cells with levothyroxine. To this end we developed nano-embedded respirable microparticles by spray drying a nanosuspension composed of levothyroxine and a hydrophilic polymer. The powder was characterized in terms of physico-chemical, toxicological and aerodynamic performance, as well as for its ability to be internalized by A549 cells and modulate their metabolic activity. The nano-embedded microparticulate drug delivery system proved to be potentially able not only to reach the deep lung but also to promote levothyroxine internalisation and mitochondria activation.

**Graphical abstract:**

**Figure:**
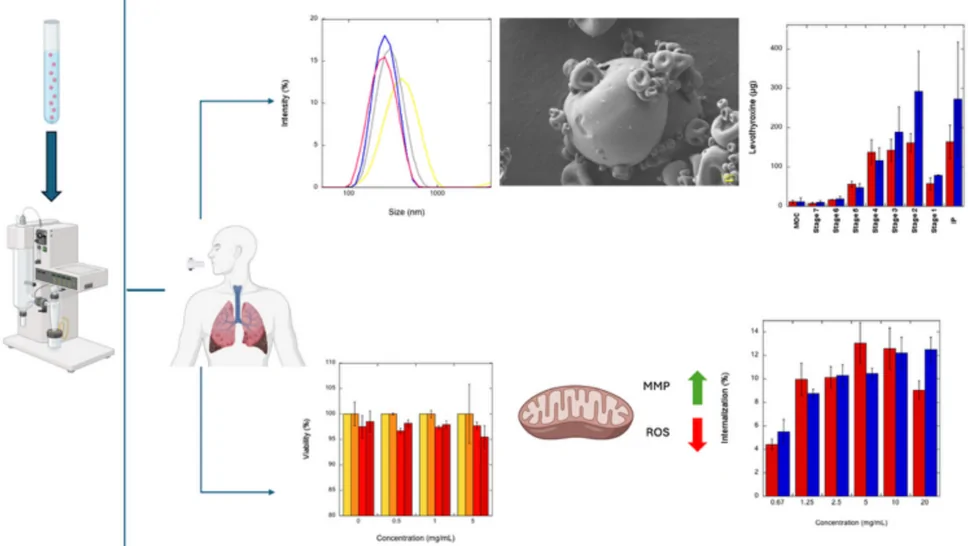

**Supplementary Information:**

The online version contains supplementary material available at https://doi.org/10.1007/s13346-026-02064-2.

## Introduction

Drug repurposing implies the identification of novel therapeutic targets for already approved drugs [1]; the interest in such a therapeutic strategy has increased significantly in the last decades, due to the possibility of shortening the development phases of new medicinal products by taking advantage of the already known toxicological data of the repurposed drug, thus offering new therapeutic alternatives to patients and business opportunities to pharmaceutical companies. Drug repurposing generally requires different dosages and administration routes to better reach the new target, for which specific drug delivery systems are often necessary [2].

Idiopathic pulmonary fibrosis (IPF) is a chronic condition in which normal lung anatomy is replaced by a mechanism of active remodeling, extracellular matrix deposition, and changes in epithelial cell phenotype. These changes in lung architecture cause an irreversible loss of lung function, eventually leading to the patient's premature death [3]. From a pathological standpoint, IPF is a complex disease that results from an interplay between repetitive alveolar epithelial cell damage, aging-related and epigenetic processes, and overproliferation of monocytes and fibroblasts combined with tissue remodeling by stimultation of macrophages and myofibroblasts.

Only two drugs are presently approved for IPF treatment: nintedanib and pirfenidone; however, besides their significant side effects, they can only slow down the disease progression without reversing the fibrotic process [4].

It is common knowledge that mitochondria play a fundamental role in lung homeostasis [5]. Their dysfunction or reprogramming also results in a mitophagy process observed in chronic lung diseases such as IPF [5]. Abnormality in mitochondrial morphology [6, 7], reduction of mitochondrial fusion process, and mitophagy [8] have been shown in the lungs of IPF patients. In these cases, the mitochondrial dysfunction has been related to a low expression of PTEN-induced putative kinase 1 (PINK1), which plays a role in the selective degradation of damaged mitochondria. The deficiency of PINK1 increases the progression of TGFβ-induced lung fibrosis [8, 9]. In the past two decades, the effect of the thyroid hormone receptor (THr) in regulating the mitochondrial fission, mitophagy and biogenesis has been demonstrated [10, 11]. In addition, Wang et al. [12] provided evidences of a possible therapeutic role of thyroid hormones in IPF treatment.

A first attempt to administer a thyroid hormone to revert the lung fibrosis was done by Yu et al. in a bleomycin-induced IPF mouse model [13] and by Li et al. in a radiation-induced lung fibrosis in mice [14]. Both studies were conducted by administering the thyroid hormone (TH) in form of nebulized a solution/suspension, affording auspicious results. However, an effective deep lung deposition can hardly be obtained by nebulization [14]. In this respect, better lung targeting can be obtained using a dry powder. In addition, in this specific case, a further level of targeting must be considered, namely the need to deliver and release the TH intracellularly. In this way, TH can reach the nucleus and interact with the THr-β, which promotes the alveolar epithelial cells type 1 differentiation and contrasts the fibrotic hyperplasia [15].

However, it must be considered that THs are poorly soluble compounds. The formation of sodium salt represents the main route to enhance TH solubility, although salification is generally related to the reduction of the drug's permeability [17]. Indeed, TH sodium salt is classified as Class 3 in the Biopharmaceutical Classification System: high solubility and low permeability [18]. This combination could be advantageous for local lung activity, with a reduction of systemic distribution. However, these conditions can be reached only in strong alkaline conditions incompatible with lung physiology.

Local delivery through nanoparticles may represent a possible solution to the issues related to the poor biopharmaceutical properties of THs [19]. Nanoparticles may be constituted by different carrier materials such as lipids [20] or polymers [21]. In this respect, Azarbayjani et al*.* evidenced, through FT-IR measurements, the capability of levothyroxine (LT4) to interact with polyvinyl alcohol (PVA) nanofibers [16], suggesting the possible exploitation of this hydrophilic polymer for LT4-containing nanoparticle production, supporting the possibility of formulating TH in polymeric nanoparticles.

Given these premises, in this work, we translate the findings derived from the above-mentioned researches into a drug delivery system designed to target the epithelial lung cell with LT4, the synthetic form of the thyroid hormone thyroxine, which is already on the market in form of oral solution, capsules or tablets for the treatment of hypothyroidism.

To this end, we developed a respirable powder constituted by nano-embedded microparticles obtained by spray drying a nanosuspension containing the active ingredient and a hydrophilic, biocompatible polymer capable of enhancing the LT4 distribution in the aqueous phase, thus improving the drug concentration in the final formulation. The overall goal was to design and develop a formulation that enables dual targeting, reaching both the deep lung and intracellular compartment of the pulmonary epithelial cells.

## Materials and methods

### Materials

LT4 powder was purchased from Sigma-Aldrich (St. Louis, Missouri, USA); ultrapure water was produced by an Arium® purification system (Sartorius, Goettingen, Germany).

Polyvinyl alcohol, PVA, 13–23 kDa (87–89% hydrolyzed) and 31–50 kDa (87–89% hydrolyzed) were obtained from Sigma-Aldrich (St. Louis, Missouri, USA). All other materials were from Sigma-Aldrich unless otherwise stated.

### Methods

#### LT4 assay by high-performance liquid chromatography

LT4 quantification was performed as described by Stewart et al. [22], using a HPLC Agilent 1200 series equipped by UV detector set at 225 nm (Agilent Technologies, Santa Clara, CA, USA). The chromatographic separation was achieved using a Zorbax C18 RP 250 mm × 4.6 mm column (Agilent Technologies, Santa Clara, CA, USA) equipped with a Phenomenex security guard (Phenomenex, Torrance, CA, USA).

The mobile phase was composed of a 50:50 v/v mixture of acetonitrile and water with 0.1% of trifluoracetic acid, pumped at 0.6 mL/min; the sample injection volume was 20µL. The LT4 retention time was 6.8 min. Each sample injection was interspersed with an injection of solvent only (methanol) to keep the sampling needle clean.

LT4 standard solution was prepared in HPLC-grade methanol at 100 µg/mL. A calibration curve was prepared by diluting the standard solution with the same solvent to get a concentration range from 100 to 0.05 µg/mL.

#### Levothyroxine solubility in water

LT4 intrinsic or apparent solubility was determined in pure water and PVA (both molecular weights) aqueous solutions at two concentrations (1% or 3.5% w/v).

An excess of LT4 was added to water or each polymeric solution and stirred for 24 h at room temperature; then the systems were centrifuged, and the supernatant was filtered with an RC membrane (0.45 µm, Sartorius, Gottingen, Germany) and analyzed by HPLC for LT4 quantification.

#### PVA – levothyroxine nano-suspension

A weighted amount of PVA was dissolved in 80 °C ultrapure water under stirring to obtain a 1% w/v solution. Thereafter, the solution was cooled down to room temperature, then LT4 at concentrations of 0.99, 1.48, 1.98, 2.91, or 3.38% with respect to the PVA weight was added as powder. The suspensions were stirred for 24 h at 25 °C, then submitted to a high-pressure homogenizer (Panda, GEA Niro Soavi S.P.A., Parma, Italy) at 400 bars for 15 cycles.

The particle size of the obtained nanosuspension was checked by dynamic light scattering, DLS, (Nanosizer ZS, Malvern Panalytical Inc., Malvern, UK) at 25 °C; the instrument was set with a material refractive index of 1.59, adsorption of 0.01, and water as dispersant phase (refractive index: 1.33, viscosity 0.8872 cP), while the measurements position was automatically set by the instrument.

#### Powder preparation

The homogenized nanosuspensions (750 mL each) were spray dried with a Mini Spray Dryer B-290 (BÜCHI Labortechnick, Flawil, Switzerland) equipped with a 0.7 mm diameter nozzle. Drying parameters were set as follows: inlet temperature 130 °C, air nebulization flow rate 742 L/h, aspiration 30 m^3^/h, and solution feed rate 3 mL/min.

Spray-dried powders were characterized in terms of LT4 content by HPLC, and particle size distribution by laser light scattering (Spraytec®, Malvern Panalytical, UK). The diffractometer was equipped with a 300 mm focal lens that can measure particles in a size range between 0.1 and 900 μm. Samples were prepared by dispersing the powder (1 mg/mL) in 0.1% w/v Span 85 (Honeywell Fluka™, Morris Plains, NJ, USA) solution in cyclohexane (Sigma-Aldrich®, Schnelldorf, Germany). Each sample was sonicated in an ultrasonic bath (Branson Ultrasonics Corporation 8510, Brookfield, CT, USA) for 5 min before the analysis to help particle separation and improve homogeneity. Each measurement was performed in triplicate.

For comparative purposes, selected powders were also submitted to dry analysis with a Mastersizer 3000 (Malvern Panalytical Inc., Malvern, UK) using the following conditions: 4 bars aspiration pressure, 60% feed rate, 30 mg sample size.

Size distribution was represented as volume diameter of the 10th (Dv_10_), 50th (Dv_50_), and 90th (Dv_90_) percentiles of the particle population. The distribution amplitude was described by the span value, calculated as [(Dv_90_ − Dv_10_)/Dv_50_].

The powder resuspendability was assessed by DLS by dispersing the powders in water at the same solid concentration as in the parent nanosuspension. The Z-potential was measured using a NaCl 1 mM solution as the dispersant phase.

The particle aggregation was further investigated by measuring the particle size distribution at different concentrations (10, 2, 1, and 0.5 mg/mL) using DLS and multi-angle DLS (Zetasizer Ultra, Malvern Panalytical Inc., Malvern, UK).

The effect of the polymer concentration was also investigated with a nano tracking analyzer (NTA) (Nanosight, Malvern Panalytical Inc., Malvern, UK) equipped with a laser wavelength of 488 nm and driven by NS XPLORER software V. 1.1.0.6. NTA measurements were performed at 25 °C in dynamic mode, pumping the sample at 3 µL/min; the instrument was set with a material refractive index of 1.59, adsorption of 0.01, and water as dispersant phase (viscosity 0.8764 cP). For each sample, 5 captures composed of 750 frames each were acquired. The instrument automatically set the camera position and contrast gain before the experiment.

The moisture content of the spray-dried powders was measured by thermogravimetric analysis using a TGA/DSC1 (Mettler Toledo, Columbus, OH, USA) driven by a STARe software V. 11.00. Samples of about 6 mg of powder were placed in alumina pan (40 µL) and heated at a rate of 10 °C/min from 25 to 130 °C under a nitrogen flux of 80 mL/min.

#### Microparticle morphology and composition

The morphology of the spray dried particles was observed by scanning electron microscopy, SEM, (Zeiss AURIGA, Zeiss, Oberkochen, Germany). The powders were dispersed on adhesive black carbon tape placed on aluminum stubs and excess powder was removed with a gentle nitrogen flow. The microscope was operated under high vacuum condition (1.16 10^–4^ Pa) with an electron beam accelerating voltage of 1.0 kV at a working distance of 4.9 mm. The low-voltage operation and the micrometer-sized dimension of the powders allowed the performance of SEM analysis without the need for surface metallization.

Energy Dispersive X-ray analysis, EDX, (Xplore 30, Oxford Instruments, Abingdon, United Kingdom) coupled with SEM was used to study the presence and distribution of LT4 on the particle surface. These measurements were performed at 10 kV electron beam acceleration voltage, to allow for X-ray emission from the elements of interest. This larger acceleration voltage required the samples to be metallized with a thin layer of Au to enable surface charge dissipation.

To better understand the microparticle morphology and their internal structure, focused ion beam, FIB, (Zeiss AURIGA Compact, Zeiss, Oberkochen, Germany) was used to slice the microparticles and observe their internal section. The Ga FIB probe settings consisted in 30 kV acceleration voltage and 50 pA ion beam current.

The dimensions of the LT4 nanostructures on the particle surface observed in the SEM pictures were determined by image analysis with the ImageJ software (U.S. National Institute of Health, Bethesda, MD, USA), using the length bar of each image as a calibration reference.

The equivalent diameter of the nanostructures was expressed as projected area-equivalent diameter(d_a_), namely, the diameter of a sphere having the same projected area as the particle's projection. The dimension distribution was calculated by measuring at least 110 particles and classifying their diameters with a progression having a ratio of √2.

#### Aerodynamic assessment

The aerodynamic performance of the powders, with the highest LT4 content, was assessed with a Next Generation Impactor (NGI; Copley Scientific, Nottingham, UK). Before the analysis to avoid particles bouncing, the plates of the impactor were coated with a solution of 2% (*w*/*v*) of Tween20 (Honeywell Fluka™, Morris Plains, NJ, USA) in ethanol and the solvent was allowed to evaporate under a hood for 40 min. The NGI was connected to a SCP5 vacuum pump (Copley Scientific, Nottingham, UK) through a critical flow controller (TPK Copley Scientific, Nottingham, UK).

The powder loaded in hypromellose size 3 capsules (Quali-V-I, Qualicaps Spain, Alcobendas, Spain) was aerosolized for 4 s with a RS01 (Plastiape, Lecco, Italy) inhaler, at a flow rate of 60 L/min to generate a pressure drop of 4 kPa through the inhaler. The flow was checked and measured using DFM 2000 Flow Meter (Copley Scientific, Nottingham UK). Due to the low active ingredient concentration, the content of three capsules, each filled with 10 mg of powder, was discharged for each experiment. LT4 deposited in each stage of the impactor from the induction port to the Micro Orifice Collector (MOC) was dissolved with 5 mL of ultrapure water and quantified by HPLC using the above-described method.

The recovery was calculated as the ratio % between the total mass of the drug collected in the whole impactor (induction port, stages 1 to 7, and MOC) and the total mass loaded in the 3 capsules.

The emitted dose (ED), namely the amount of drug discharged from the device, was determined by weighing the device loaded with the filled capsule before and after the aerosolization.

LT4 collected in each stage was used to build a cumulative undersize percentage mass distribution plot with respect to the cut-off diameters of each stage. In particular, the mass median aerodynamic diameter (MMAD) and the geometric standard deviation (GSD) were calculated from the plot of the cumulative undersize percentage of the collected drug (in probit scale) versus the cut-off values of each stage in log scale according to European Pharmacopoeia specifications [23]. This plot also allowed for the determination of the fine and extrafine particle fraction, corresponding to the percentage mass of the drug emitted having an aerodynamic diameter lower than 5 and 2 µm respectively. These values were used to estimate the fine particle dose, namely the micrograms of LT4, which was estimated to reach the lower respiratory airways. Each test was carried out in triplicate.

#### Cell culture

In vitro experiments were carried out using a continuously growing cell line of human lung origin A549 (adenocarcinoma human alveolar basal epithelial cells) purchased from the American Type Culture Collection (Manassas, USA). Cells were grown in low glucose Dulbecco's Modified Eagle's Medium (DMEM) (Biowest, Nuaillé, France, Batch MS01TA) with 1% of non-essential aminoacids100X (Corning, NY, USA, Lot 25,621,008), 1% penicillin, streptomycin solution 100x (Corning, NY, USA, lot.30002370) and supplemented with 10% Fetal Bovine Serum, FBS Gibco standard (Gibco™, Thermo Fisher Scientific, Waltham, USA, Lot. 2556826RP) at 37 °C in 5% CO_2_ controlled environment. Cells were split at 80% of confluence, assessed by microscope observation.

#### Cytotoxicity assay

The toxicity of the raw materials (LT4 and PVA at both molecular weights) and of the spray-dried formulations was assessed by MTT assay [24].

LT4 was dissolved in DMSO (30 mg/mL) and then diluted in cell culture medium at 9 different concentrations (0.11, 0.23, 0. 46, 0.93, 1.87, 3.75, 7.5, 15 and 30 µg/mL).

PVA was dissolved at 10% w/v in phosphate buffer saline (PBS) (Corning, NY, USA) and then diluted in cell medium to get 1%, 0.5%, 0.25% and 0.125 w/v concentrations; for the lower MW PVA the concentration 0.062 w/v was also tested.

The formulations produced with the higher concentration of LT4 (S5 and S10) were tested by resuspending the powders directly in cell medium at 10.1 mg/mL and then diluting with the medium to get 5.05, 2.52, 1.26, 0.63, 0.31, 0.15, 0.78, and 0.03 mg/mL concentrations.

For the toxicity studies 10^4^ cells/well were plated in a polystyrene 96 multiwell flat bottom plate (Primo® multiwell plate, Euroclone S.p.A., Milan, Italy) and cultured until the formation of a monolayer. One hundred microliters of the raw material solutions or formulation suspensions were applied on the cell monolayer for 4 h, and the cell viability was assessed by MTT assay. For the latter, 100 µL of thiazolyl blue tetrazolium bromide (98%, Sigma Aldrich, Saint Luis, USA) at a concentration of 0.5 mg/mL in PBS, was applied to each well; the solution was removed after 3 h of incubation at 37 °C. The formed crystals were dissolved by the addition of 100 µL of DMSO. The absorbance of the obtained solution was measured at 570 nm using a Spark multimode microplate reader (Tecan, Mannendorf, Switzerland).

A further MTT assay was performed on the formulations to assess their long-term effect (5, 24, 48 h) using three different concentrations: 0.5, 1, and 5 mg/mL.

The effect of the treatments on cell metabolic activity and viability was expressed as a percentage ratio with respect to the medium-treated cells (control).

#### Cell proliferation and long-term viability

The effect of the formulation on the cellular growth and viability was also investigated using a 4', 6-diamidino-2-phenylindole (DAPI) (Sigma Aldrich, Saint Luis, USA) labelling. Cells were plated at 25 × 10^3^ in a 12-wells plate and incubated overnight before being treated with both the formulation at three different concentrations (5, 1, 0.5 mg/mL).

The effect of treatments on proliferation and viability was evaluated after 24, 48, and 72 h by flow cytometry as described below. Cells were covered with a thin layer of trypsin solution (0.25% w/v) (Corning, NY, USA) to detach them from the well and resuspended in 600 µL of medium. Half of the sample was added with DAPI (5µL for each sample) to label dead cells before the analysis.

As a positive control of cell death, a well of non-treated cells was trypsinized, resuspended in 300 µL of medium. Thereafter, half of this sample was heated at 65 °C for 5 min, then mixed with the other half and supplemented with 5µL of DAPI to obtain 50% of DAPI-positive cells. The measurements were carried out by using a 405 nm laser for the excitation of DAPI and the emission was recorded with a 450/50 nm filter.

#### Cellular uptake

The cellular uptake of LT4 both as solution and reconstituted nanosuspension, was evaluated in the A549 cell line. The two spray dried formulations were suspended in the medium at 0.675, 1.25, 2.5, 5, 10, 20 mg/mL concentration.

As a control PVA at the two MW was dissolved in ultrapure water, then diluted in the medium to get a final concentration of 10 mg/mL. Finally, LT4 was dissolved in DMSO (30 mg/mL) and then diluted in cell culture medium to get a final concentration of 15 µg/mL; This concentration value represents the highest achievable in the medium, without phase separation.

Cells were cultured in a 24-well plate to obtain a homogenous monolayer and cells were then treated for 1, 2, 4, 6, 8, and 24 h, with 1 mL of either formulation nanosuspension or solutions of the raw materials.

The LT4 intracellular content was quantified after cellular lysis. Cells were detached from the plate by scraping, resuspended in 1 mL of methanol/well, and then centrifuged at 4 °C, 1000 g for 30 min. The obtained supernatants were filtered (0.45 µm) and injected in the HPLC for quantification of the active ingredient. Each treatment was carried out in 3 independent replicates.

#### Flow cytometry

Flow cytometry experiments were performed on MacsQuant Analyzer 10 (Miltenyi Biotec GmbH, Bergisch Gladbach, Germany) on cells kept on ice before the analysis. The emission channels and filters used are summarized in Table S1. The data were analyzed using FlowJo software v10 (BD Life Science, New Jersey, USA). The comparison between the experimental conditions for the mitochondrial membrane potential was made by two-way analysis of variance (ANOVA) followed by Tukey's post hoc test for multiple comparisons, using Prism 8.0.1 (GraphPad software, San Diego, CA, USA) with a significance level of ɑ = 0.05 (*P* < 0.05).

The effect of the formulation on the reactive oxygen species (ROS) production capacity of cells was explored using the ROS-sensitive probe, dihydrorhodamine 123 (DHR, D105, St. Louis, Missouri, USA). DHR stock solution was prepared in DMSO at 10 mM.

The measurements were carried out by using the 488 nm laser for the excitation of DHR and the emission was recorded with the 525/50 nm filter.

A459 cells were seeded at 50 × 10^3^ cells/cm^2^ in a 12-well plate. The day after seeding, cells were treated with 0.5, 1, or 5 mg/mL of the reconstituted nanosuspensions in medium. The ROS production was measured after 5, 24, 48, and 72 h. Untreated cells were used as a control to define the basal oxidative stress stemming from the manipulation. For the analysis, cells were trypsinized and resuspended in medium supplemented with 5 µM DHR; after 15 min of incubation in the dark at 37 °C, cells were analyzed with a cytofluorimeter, counting 15 × 10^3^ events of interest for each sample.

Hydrogen peroxide (H_2_O_2_) was used as a positive control of ROS production to induce the complete oxidation of DHR. After DHR incubation, 1µL/mL of H_2_O_2_ was added to the detached cells and incubated at 37 °C for 15 min.

The mitochondrial membrane potential was evaluated using tetramethylbenzimidazoylcarbocyanine iodine (JC-1) [25]. JC-1 dye exhibits potential-dependent accumulation in mitochondria, indicated by a green fluorescence emission at ~ 529 nm for the monomeric form of the probe, which shifts to red (~ 590 nm) with a concentration-dependent formation of red fluorescent JC-1-aggregates (Fig. 4). Consequently, the decrease in the red/green fluorescence intensity ratio is taken as a measure of mitochondrial depolarization.

A549 cells were seeded at 25 × 10^3^ cells/cm^2^ in a 12-wells plate, and treated with 0.5, 1, and 5 mg/mL of formulation. 24 h after seeding. As described before, the plates were analyzed after 5, 24, 48, and 72 h of incubation; untreated cells were analyzed to define the physiological mitochondrial membrane potential.

For the analysis, cells were trypsinized and washed with PBS, then resuspended in JC-1 solution and incubated at 37 °C for 20 min. After the incubation, cells were washed twice with ice-cold PBS and finally resuspended in PBS and analyzed by cytofluorimetry: the measurements were performed using a 488 nm laser for the excitation and the two different emission filter: 525/50 and 585/40 for the green (monomeric state) and red (aggregate state) signals respectively.

As a positive control, cells were co-treated for 20 min with JC-1 and with carbonylcyanure m-chlorophenylhydrazone (CCCP), which represents an established positive control for the mitochondrial membrane potential disruption [26].

## Results and Discussion

### Levothyroxine intrinsic or apparent solubility in water and in PVA aqueous solutions

**Fig. 1:**
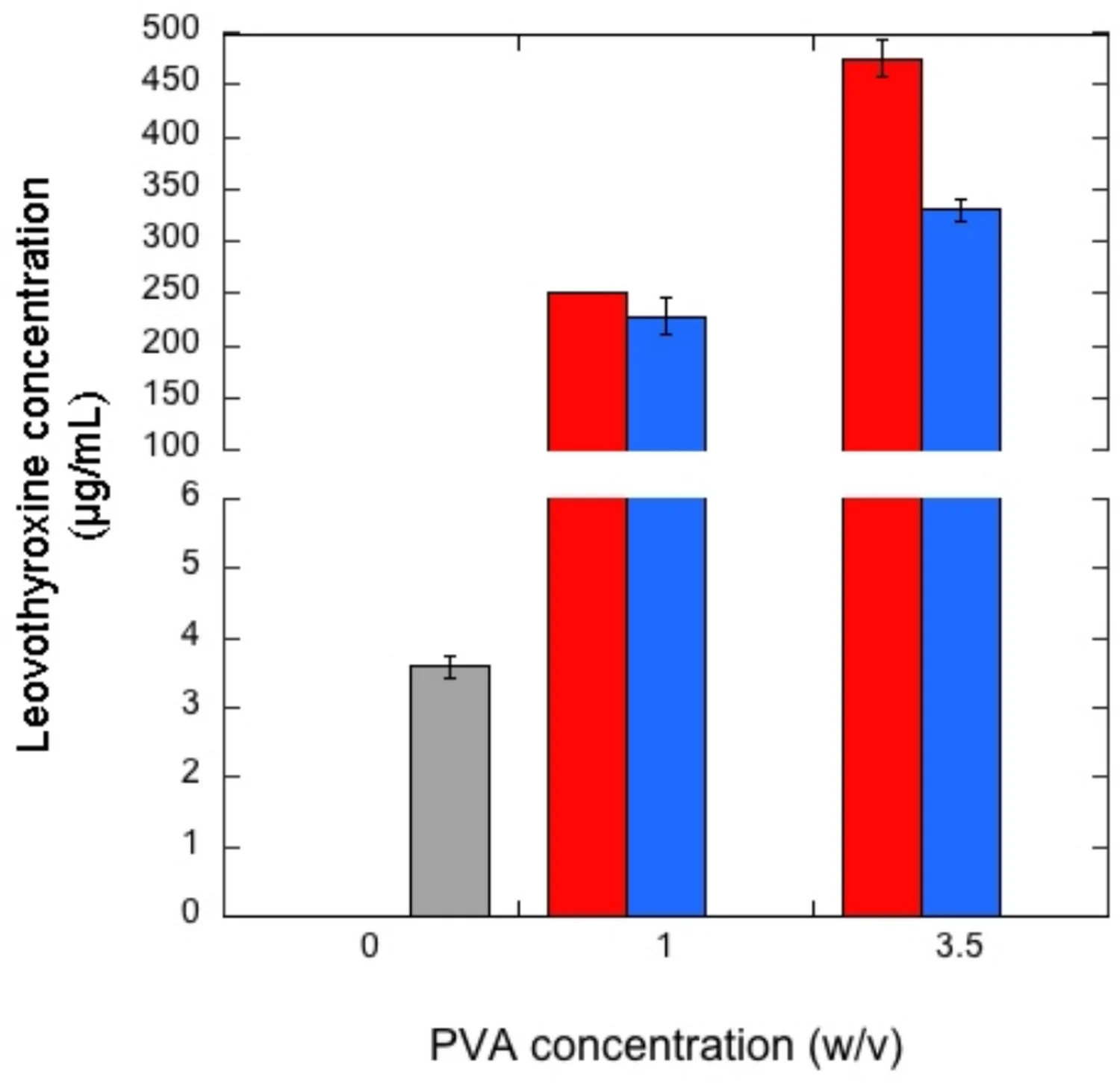
LT4 equilibrium concentration at 25 °C, in water (grey column), in PVA 13–23 kDa aqueous solution (red columns), and PVA 31–50 kDa aqueous solution (blue columns). The black bars represent the standard deviation (*n* = 3)

Figure 1 presents the LT4 equilibrium concentration, in aqueous solutions in the presence or absence of PVA at two MWs and concentrations.

The intrinsic solubility in water was 3.59 ± 0.15 µg/mL and in good agreement with the literature data [27, 28]. In the presence of PVA, the LT4 apparent solubility increased by about 70-fold for the lower polymer concentration and over 90-fold for the higher concentration compared to pure water.

It is important to note that upon establishment of the equilibrium between LT4 and the polymer solution, the liquid phase became opalescent, indicating the spontaneous formation of a colloidal system, which remained the same even after filtration with a 0.45 µm regenerated cellulose membrane (Fig. S1 in Supplementary materials). This phenomenon was likely related to an interaction between LT4 and PVA in the aqueous phase involving the formation of hydrogen bonds [29]. This unexpected effect was considered a favorable starting point for developing a particulate drug delivery system containing a suitable LT4 dosage.

### PVA – levothyroxine colloidal system

Based on the results reported in Fig. 1, it was acknowledged that the higher polymer concentration (3.5%w/v) could better enhance LT4 apparent solubility with respect to the lower one. However, the observed increase (1.9 and 1.4 folds for the low and high polymer MW, respectively) was considered insufficient to justify the associated 3.5-fold increase in polymer content (3.5 folds) which would result in an excessive excipient load and potentially overload the lungs. In fact, in formulation sciences, particularly in the pulmonary field, it is a good practice to minimize excipient level to ensure that patients to inhale the smallest possible amount of non-active ingredients [30]. For this reason, the polymer concentration was set to 1% w/v while different LT4 concentrations (from 0.99 to 3.38% of the polymer weight) were tested to assess the spontaneous formation of the colloidal dispersion, which was prepared as self-assembling systems by adding LT4 powder to the PVA solution. After 24 h of stirring, all systems appeared opalescent with a particle average size, determined by DLS, around 600 nm and a polydispersity index > 0.4 irrespective of the LT4 concentration and PVA MW tested.

A high-pressure homogenization step afforded a reduction of about 200 nm in particle size that leveled around 400 nm, and sharper polydispersity (~ 0.3), again irrespective of drug concentration and polymer molecular weight. For this reason, in the subsequent phase of respirable powder preparation, only the higher LT4-containing formulation (3.38% w/w) was considered for both the low and high polymer MW.

### Powder characterization

The two colloidal suspensions were then spray dried to obtain a dry powder suitable for pulmonary administration. Powders were obtained in both cases with a yield > 60%.

The two powder obtained with the low and high MW PVA, coded as S5 and S10, respectively, were first submitted to laser lifgt diffraction measurements for particle size distribution determination.

Table 1 shows the DV_10_, DV_50_, DV_90,_ and the amplitude of the population (Span) of the two spray dried powders determinedby laser diffraction in both wet and dry mode.

**Table 1:** Particle size distribution of the produced powders, mean values ± standard deviation (*n* = 3)

Mode	Sample	DV10 (µm)	DV50 (µm)	DV90 (µm)	Span
Wet	S5	1.97 ± 0.10	5.52 ± 0.15	14.73 ± 0.47	2.31 ± 0.14
Wet	S10	2.82 ± 0.51	9.18 ± 1.87	19.08 ± 2.83	1.77 ± 0.12
Dry	S5	1.55 ± 0.14	4.28 ± 0.07	15.90 ± 1.27	3.40 ± 0.23
Dry	S10	1.37 ± 0.02	4.09 ± 0.05	17.50 ± 1.97	3.95 ± 0.43

In wet conditions,the higher MW PVA (S10) allowed the formation of larger particles with a narrow size distribution. The particle size distribution of two formulations was also determined in dry mode to avoid possible biases stemming from the incomplete dispersion of the powder in the liquid cyclohexane (used as a non-solvent in the sample preparation). As shown in Table 1, the particle size of the two powders measured in dry mode substantially overlapped with a significant, although not dramatic, reduction with respect to the data obtained in wet mode, especially for the high MW PVA-containing powders.

These particle size distributions suggest the suitability of these powders for pulmonary delivery as they presented a median diameter lower than 5 µm, although expressed as geometric rather than aerodynamic equivalent diameter.

The two powders' residual humidity (moisture) was assessed by TGA and resulted 1.7 and 5.3% (w/w) for the low and high MW PVA-containing powder. This was not surprising considering the polymer's hydrophilic nature, which implies an increase in residual moisture with the increase of the length of polymer chains bearing OH groups.

The LT4 content of the powders as determined by HPLC and taking into consideration the moisture content, was 3.26 ± 0.18% (w/w) for the low MW PVA-containing powder and 3.72 ± 0.23% (w/w) for the high MW PVA-containing powder, not statistically different from the theoretical values of 3.38% (w/w) (*p* = 0.48 and 0.12 respectively).

### Microparticles morphology

**Fig. 2:**
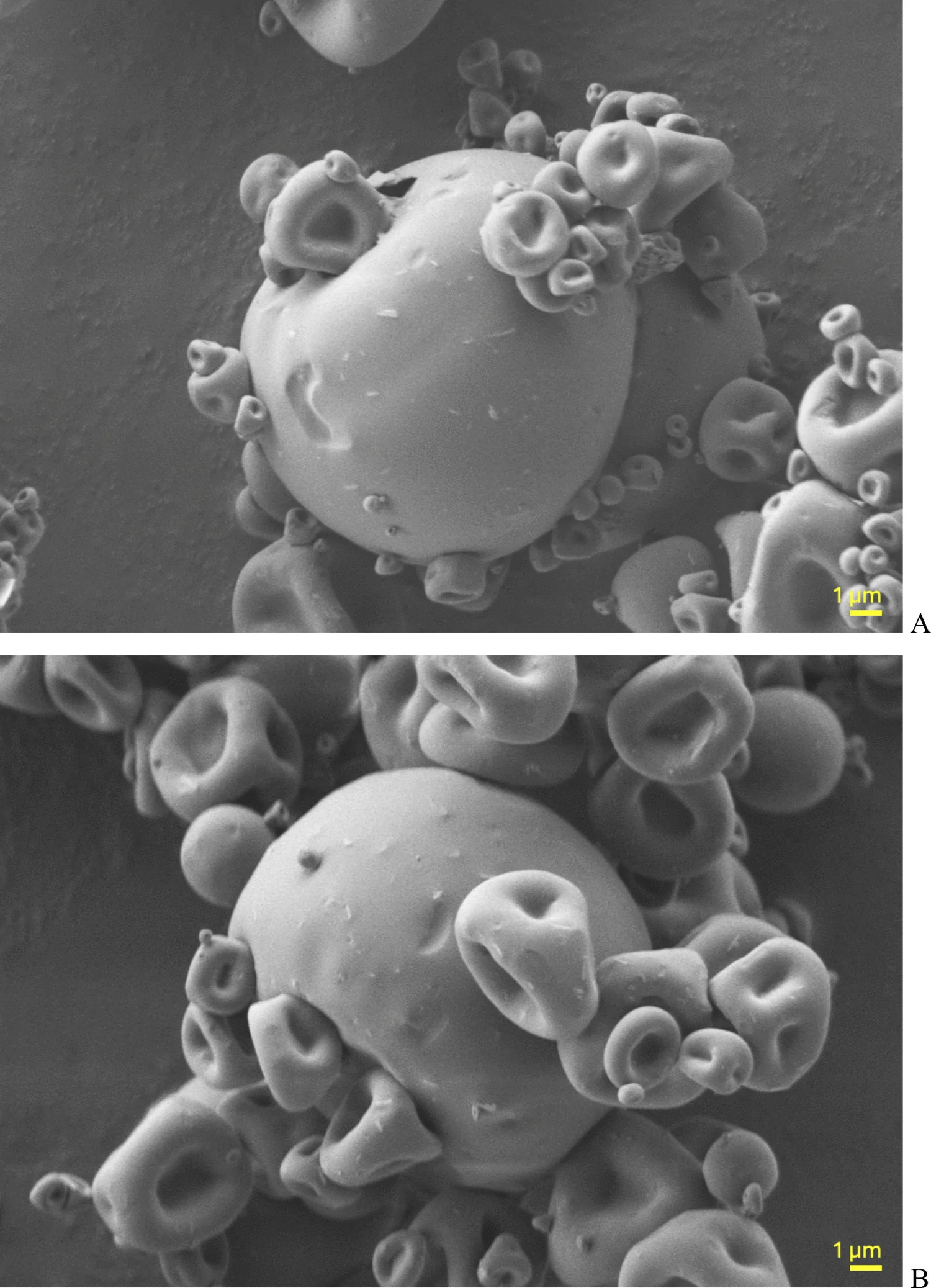
SEM images obtained at 10000X magnification from the powders prepared with PVA 13–21 kDa (**A**) and PVA 31–50 kDa (**B**) with, 3.38% w/w of LT4

Figure 2 shows the particle morphology of the produced powders. In both cases, aggregates composed of particles with varying sizes are clearly visible. A comparison between the powders prepared with the two PVA molecular weights reveals no significant differences in particle morphology or size.

Particles display an almost spherical shape with the presence of concave regions on the surface; this peculiar morphology is likely due to the particle collapse due to the vacuum applied in the measuring chamber, suggesting that empty shells constituted the particles.

**Fig. 3:**
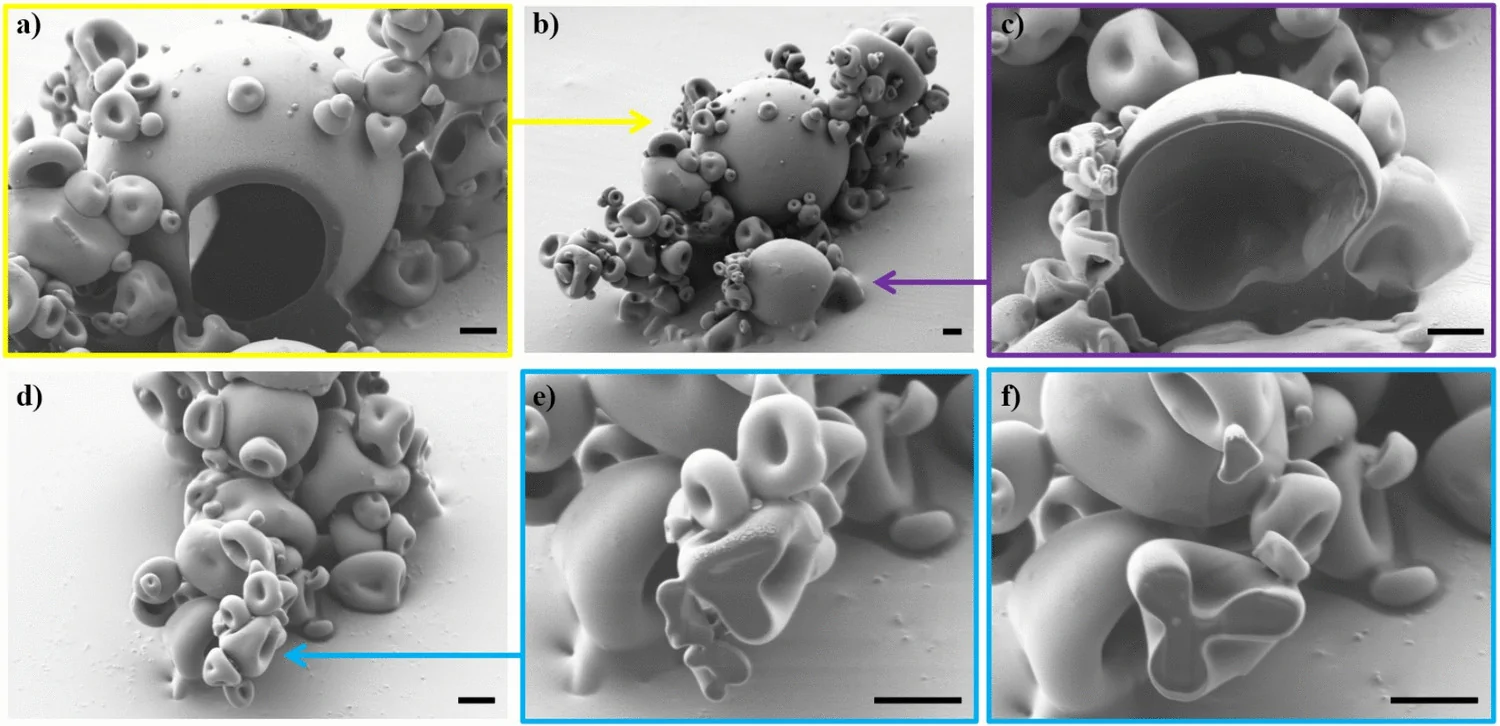
Tilted SEM images of (**b**, **d**) pristine microparticles, (**a**-**c**) FIB-cut spherical-shaped particles and (**e**-**f**) FIB-cut particles with concave surfaces. Coloured arrows and frames connect the pristine particles with the respective FIB-cut cross-sections. Images **e **and **f** represent successive FIB-cut cross-sections of the same area. All black bars correspond to 1 µm marker, since images magnification spans from 10000 to 50000X

This interpretation was confirmed by focus ion beam (FIB) section images. As representative examples, Fig. 3 shows three cross-sectioned particles, some spherical and some collapsed, where the internal part is visible. The still spherical-shaped particle appears empty inside and is characterized by a shell of a few hundred nanometers thick. The collapsed microparticle, on the contrary, presented a compact inner structure.

**Fig. 4:**
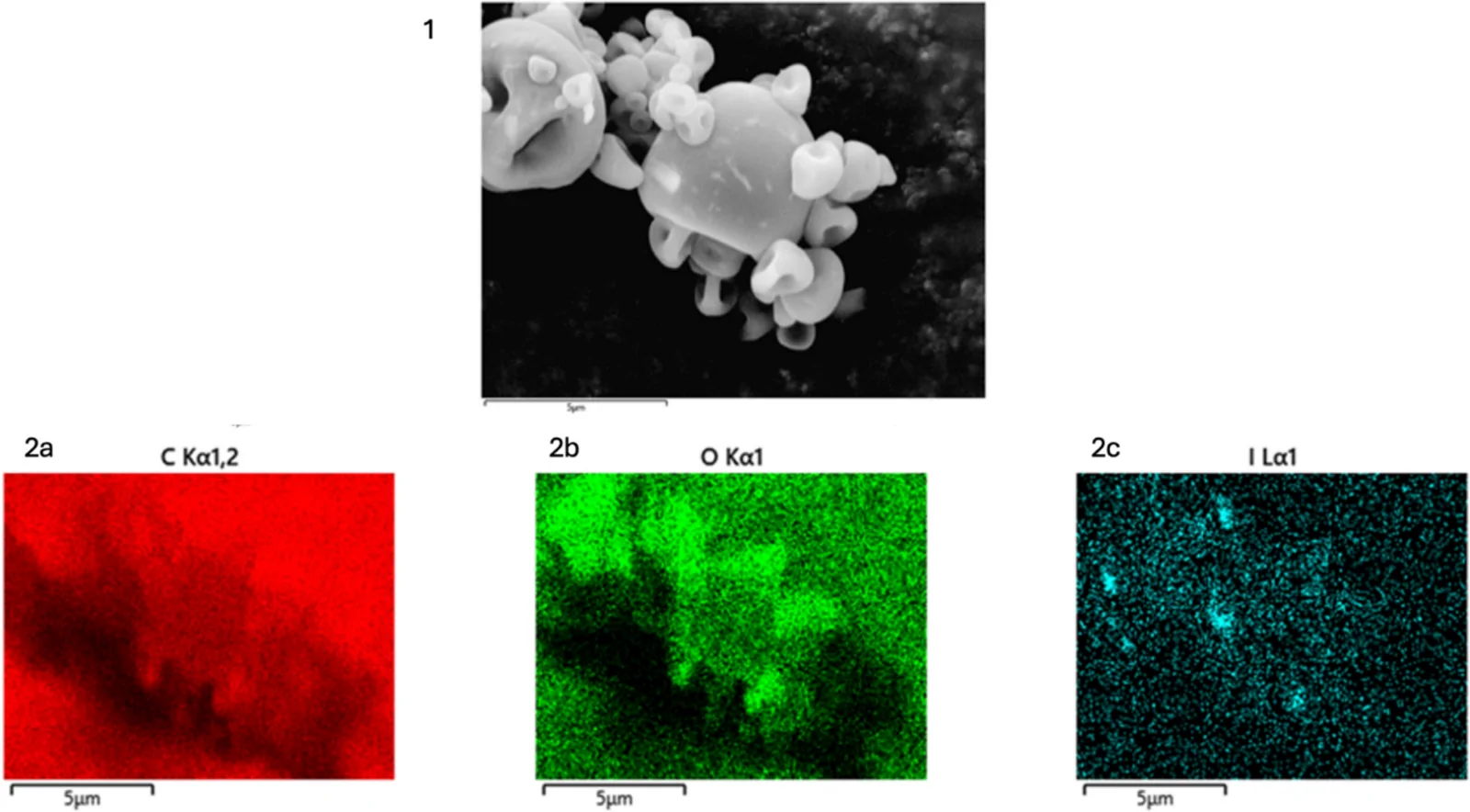
EDX analysis of S10 microparticles. Panel 1 shows the SEM acquisition of the particles selected for the elemental mapping, while the Panels 2a, b, c, show the EDX mapping of carbon (red) oxygen (green) and iodine (light blue) respectively

**Fig. 5:**
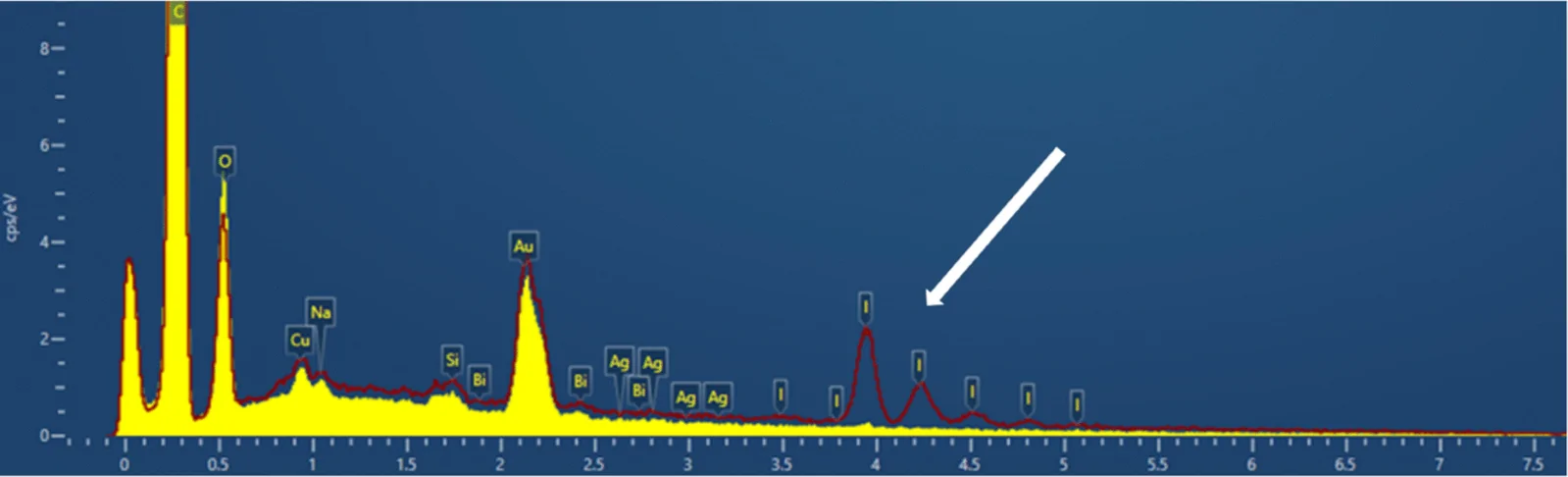
EDX spectra of the microparticle smooth surface (yellow) and the of the sub-micron structure (red)

Interestingly, the presence of small submicron-sized and needle-shaped structures with light gray contrast can be appreciated both onto the microparticle surface (Fig. 2) and inside the particle cross-section (Fig. 3c). Considering the relative ratio between the two components of the formulation, we hypothesized that LT4 particles could constitute these submicron structure. To confirm this hypothesis, energy dispersive X-ray (EDX) analysis was performed by scanning the particle surface to evaluate the elemental particle composition in terms of three diagnostic atoms, namely carbon, oxygen and iodine, the latter being present only in the LT4 chemical structure. Figure 4 shows a map of the atomic distribution on the particle surface. While, as expected, carbon and oxygen are ubiquitous, the iodine could only be detected in discrete areas which coincided with the submicron structures observed in Fig. 4, Panel 1. To investigate this aspect better, a EDX point analysis was performed to measure possible differences in iodine concentration between the smooth surface of the microparticles (areas without sub-micron structures) and the surface of the sub-micron structures. Figure 5 compares the EDX point analysis obtained on the surfaces of the smooth region of the microparticle (yellow) and the sub-micron particle (red and arrow), clearly showing that iodine is detectable only in the latter one. This demonstrates that the sub-micron particles are mainly composed of LT4, whereas the absence of iodine on the surface of the smooth region of the microparticles suggests that they are essentially composed of PVA.

### Sub-micron structure size distribution

An estimate of the size distribution of the observed sub-micron structures was done by image analysis. The obtained data are reported in Fig. S2 (supplementary materials) as cumulative undersize distribution.

The median (D50) diameter was 135 and 138 nm for the low and high PVA MW formulation, respectively, with a relatively narrow distribution (Span value 1.86 and 0.94, respectively).

These results along with those of the SEM analyses indicate that the solid particles obtained by spray drying are constituted of shells of PVA embedding LT4 nanocrystals. Thus, since PVA is a soluble polymer, these microparticles should afford a nanosuspension of LT4 particles with an average size around 130–140 nm upon dispersion in water.

The ability of the powders to reconstitute the nanosuspension upon contact with an aqueous medium is a key step for cell internalization once the powder comes in contact with the lung epithelium. To this end, the two powders were resuspended in water at the same solid concentration as before the spray drying, affording a DLS measurement of 450.4 ± 7.39 nm (P.I. = 0.326 ± 0.039) for the powder S5 and 473.2 ± 1.53 nm (P.I. 0.236 ± 0.021) for the powder S10, indicating a substantial reversibility of the drying process without the formation of non-dispersible aggregates. These data are somehow inconsistent with the observation made on SEM images and can be explained considering a possible role played by the polymer in the observed nanoparticle hydrodynamic dimension obtained by DLS. This may be ascribed to the formation of polymeric chains supramolecular structures in suspension or aggregation phenomena stemming from the absence of surface charge on the particle. Therefore, the DLS measurements were repeated on diluted samples, both in back scattering and in multi-angle mode, to account for the non-perfectly spherical shape of the particles and coupled with the determination of the particles' Z potential.

The Z-potential measurement at 1 mg/mL concentration indicated that the surface charge was practically negligible (−1.55 ± 3.46 and −2.56 ± 4.11 mV for low and high MW formulation respectively).

Fig. S3 (supplementary materials) reports the hydrodynamic size distribution obtained in back-scattering mode at different concentrations of the formulation (10, 2, 1, and 0.5 mg/mL) both for S5 and S10. The particle size distribution decreased with the decrease of the concentration, with the median diameter diminishing from 400 to 220 nm for S5 and from 400 to 255 nm for S10.

Similar data were obtained by MaDLS (Tables S2 and S3 in supplementary materials) that evidenced the progressive reduction of the size distribution with the increasing dilution.

These data strongly suggest an effect exerted by PVA upon particle resuspension. The polymeric chains self-organized around the LT4 nanoparticles at high concentration of the formulation, while polymer disentanglement occurred upon dilution allowing for the measurement of the sole LT4 nanoparticles.

To further support this interpretation, the size distribution of the formulations after resuspension was assessed also through Nano Tracking Analysis which measures the actual particle dimension rather than the hydrodynamic diameter. The obtained DV_10_, DV_50_ DV_90_ values of the measured samples diluted at 0.5 mg/mL are summarized in Table S4 in the Supplementary materials. It can be appreciated that, considering the different measurement techniques, that data are in quite good agreement with those reported above and obtained by SEM imaging analysis.

It is worth underscoring that said particle size distribution is potentially suitable for the intracellular delivery of LT4 [31] and for macrophages clearance escape [32].

Table S4 report also the data obtained upon resuspension of the powders stored for 24 months at room temperature in amber vials with rubber stopper; notably the storage did not significantly affected the nanoparticle size distribution.

### In vitro respirability assessment

**Fig. 6:**
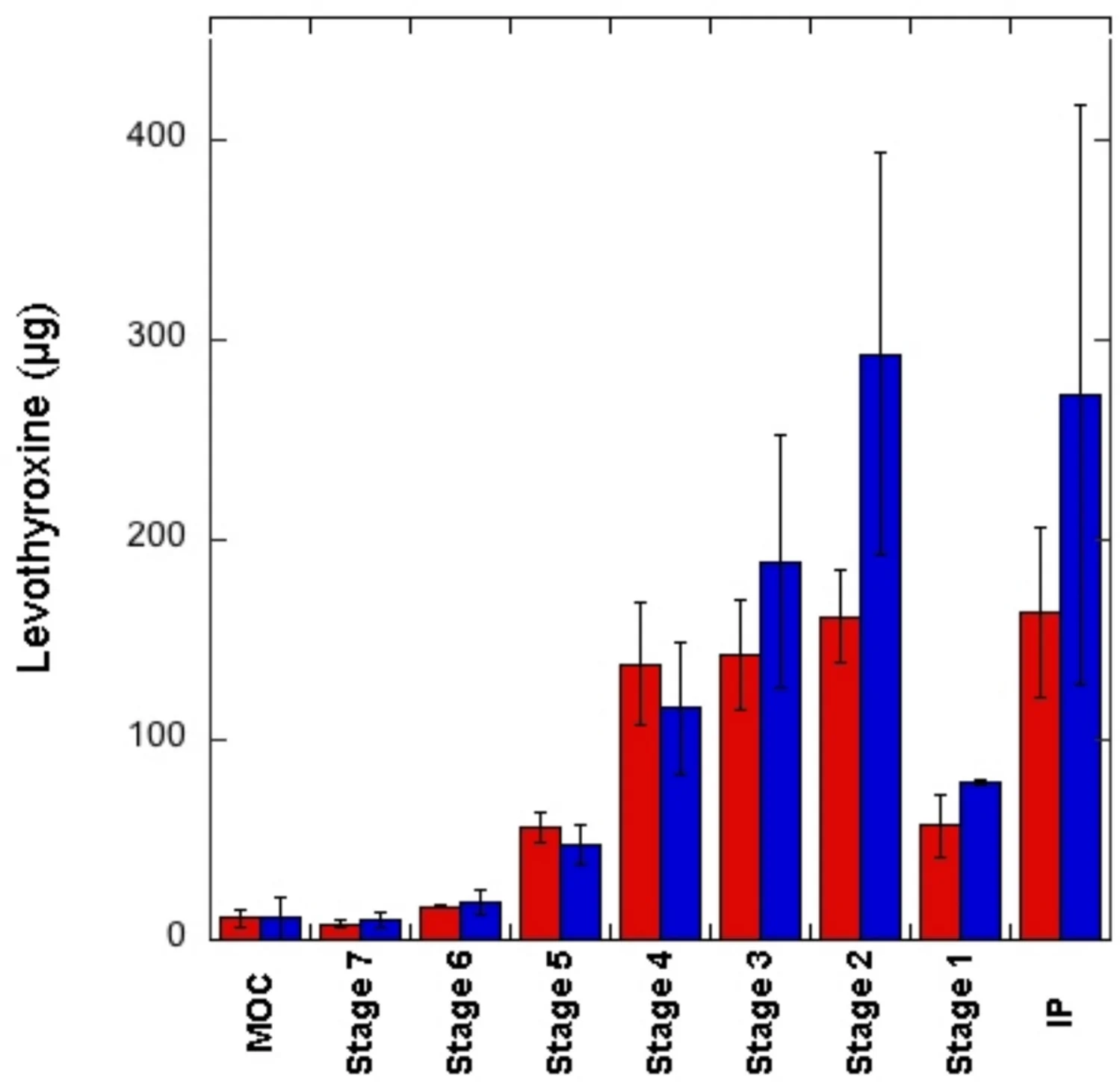
LT4 mass distribution in the NGI. In red formulation S5 and in blue formulation S10. The bars represent the standard deviation (*n* = 3)

To obtain LT4 intracellular delivery in the lung epithelium, the powder deposition in the alveolar region represents a *sine qua non* condition. To this end the in vitro deposition of the powder upon aerosolization with an RS01 inhaler was assessed with a NGI apparatus (Fig. 6).

The two powders showed a similar deposition trend; however, S10 presented a higher variability in the induction port and stage 2. This high variability may be due to cohesive interaction, which plays a significant role in the high molecular weight PVA.

Table 2 shows the comparison between the two formulations. Both powders presented high emitted doses (83.65 ± 9.64, and 89.54 ± 3.39 for samples S5 and S10, respectively).

**Table 2:** Aerodynamic parameters of two LT4-containing formulations prepared with low and high molecular weight PVA, mean values ± standard deviation (*n* = 3)

	Formulation S5	Formulation S10
*Emitted dose (%)*	83.65 ± 9.64	89.54 ± 3.39
*MMAD (µm)*	3.50 ± 0.15	4.47 ± 0.18
*GSD (µm)*	2.12 ± 0.10	2.24 ± 0.07
*Fine particle fraction (%)* < *5 µm*	75.04 ± 0.98	70.38 ± 0.78
*Extra-fine particle fraction (%)* < *2 µm*	59.40 ± 0.23	56.74 ± 0.17

MMAD was lower for the formulation prepared with the 13–23 kDa PVA (3.50 ± 0.15 µm) compared to the 31–50 kDa (4.47 ± 0.18 µm), although both were below 5 µm, which is considered a threshold value for peripheral lung deposition.

Finally, the fine particle fraction was higher than 70% for both powders, affording a fine particle dose of around 237 µg/capsule. Interestingly, the extra-fine particle fraction (aerodynamic diameter < 2 µm) was close to 60% indicating that that the dose potentially deposited in the alveolar region was around 220 µg per capsule.

It is worth underscoring that after 2 years of storage at room temperature, the MMAD, the fine particle fraction, and the extrafine particle fraction were superimplable with the value obtained after the preparation, while a small, and statistically significant (*p* < 0.01) increase in the emitted dose was observed (Table S5 in Supplementary materials).

### Effect of formulations on cell viability and proliferation

**Fig. 7:**
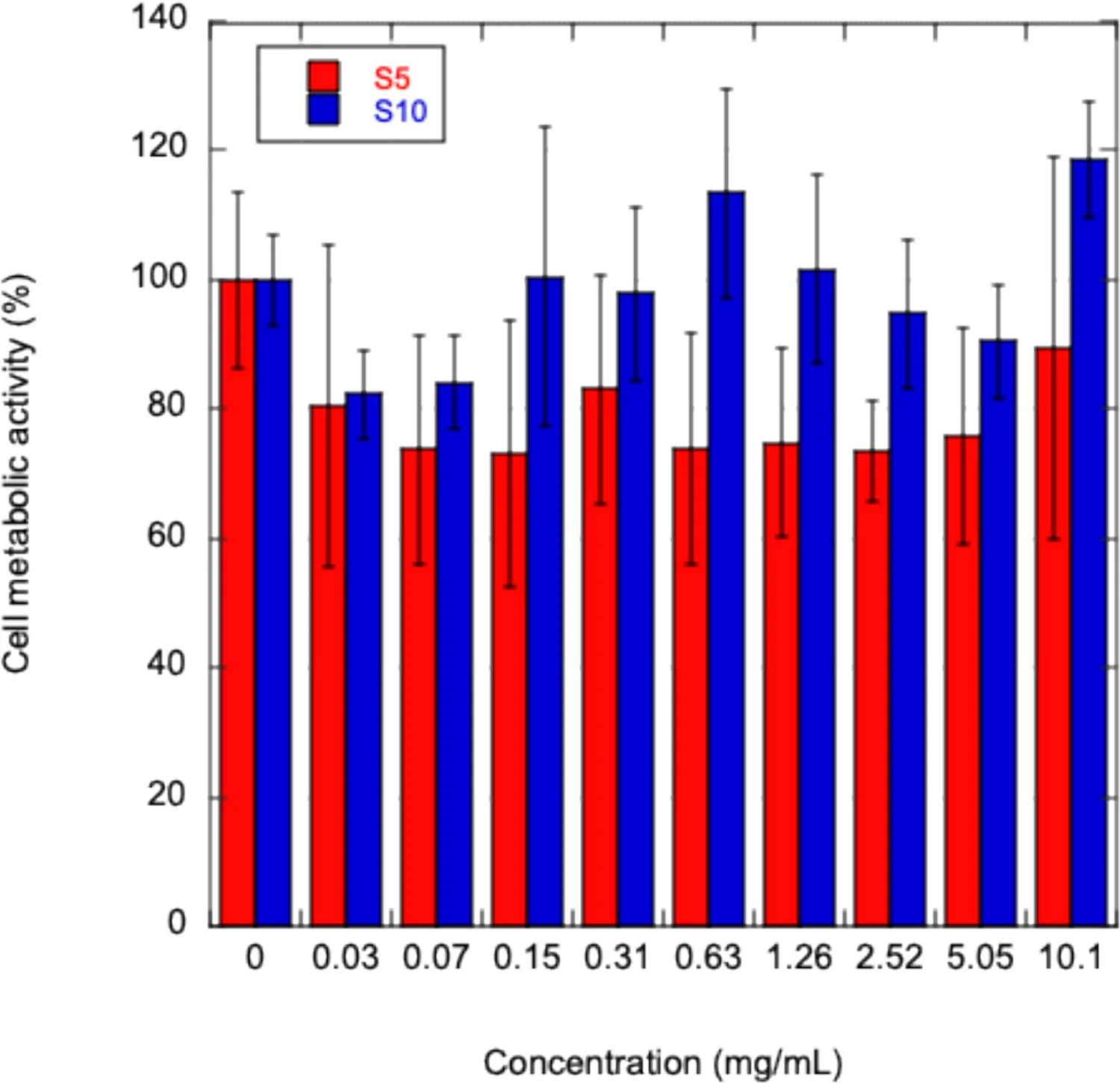
A549 cell metabolic activity (% of control) (viability) after exposure to two LT4 nanoformulations; the formulations concentrations were: 0.03, 0.07, 0.15, 0.31, 0.63, 1.26, 2.52, 5.05, 10.1 mg/mL. In red sample 5 (S5) and in blue sample 10 (S10), point 0 represents the control, namely the non-treated sample. The bars represent the standard deviation (*n* = 5)

First, the raw materials' toxicity was evaluated in a A549 cell line after 4 h exposure to solutions of LT4 and both PVA molecular weights. In all the cases, the mitochondrial metabolic activity was near or above 80% (Fig. S4 and S5 in supplementary materials). Cell viability did not change significantly with the increased LT4 concentration in the tested range, levelling around 100%. Similar results were obtained with the PVA, confirming the absence of cytotoxicity of this polymer in the tested concentration range [33]. Thereafter, the formulations S5 and S10 were tested: as shown in Fig. 7, all the samples, at all the tested concentrations, presented a viability above 70%, which represents the reference threshold indicating the non-toxicity of a compound/formulation [34].

The viability recorded for formulation S5 was statistically lower than that for formulation S10 at formulation concentrations 0.15, 0.31, 0.63, 2.52, and 10.1 mg/mL.

To further evaluate the effect of the treatment with the formulations, the MTT assay was performed by exposing the cells to the formulation at three different concentrations (namely 0.5, 1, and 5 mg/mL) for 5, 24, and 48 h.

**Fig. 8:**
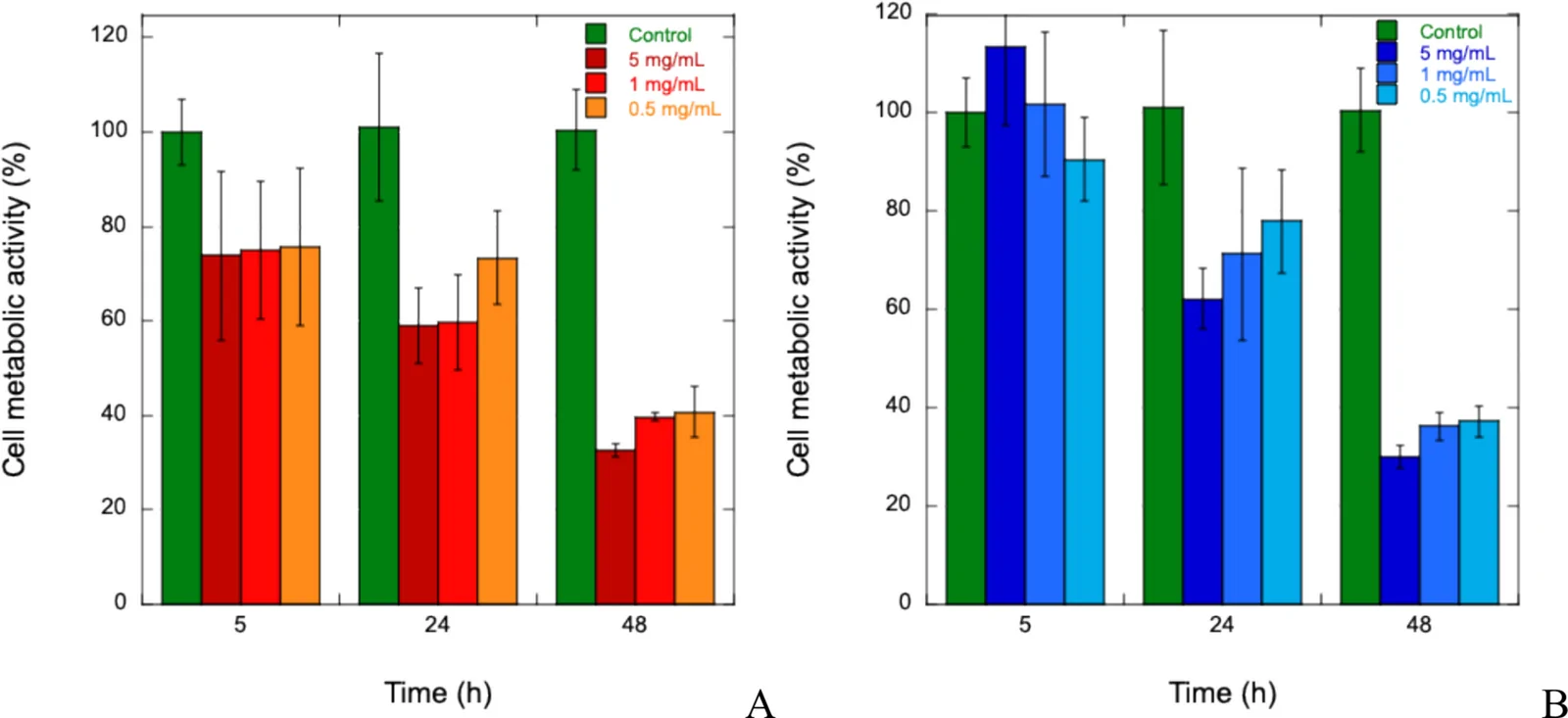
A549 cell viability, measured by MTT assay, after exposure with three different concentrations of S5 (**A**) and S10 (**B**), in green the viability of untreated cells. The black bars represent the standard deviation (*n* = 5)

Figure 8 shows the cell metabolic activity (viability) measured by MTT in the long-term exposition. After 5 h of exposure, the cells treated with S5 afforded 70% viability; after 24 h the viability was still close to 65%, whereas, after 48 h a significant drop was observed.

Differently from S5, the cells treated with S10 showed excellent viability (over 90%) after 5 h; after 24 h the viability decreased between the 60% and 70%. Similarly to S5 after 48 h the viability dropped to 30%.

**Fig. 9:**
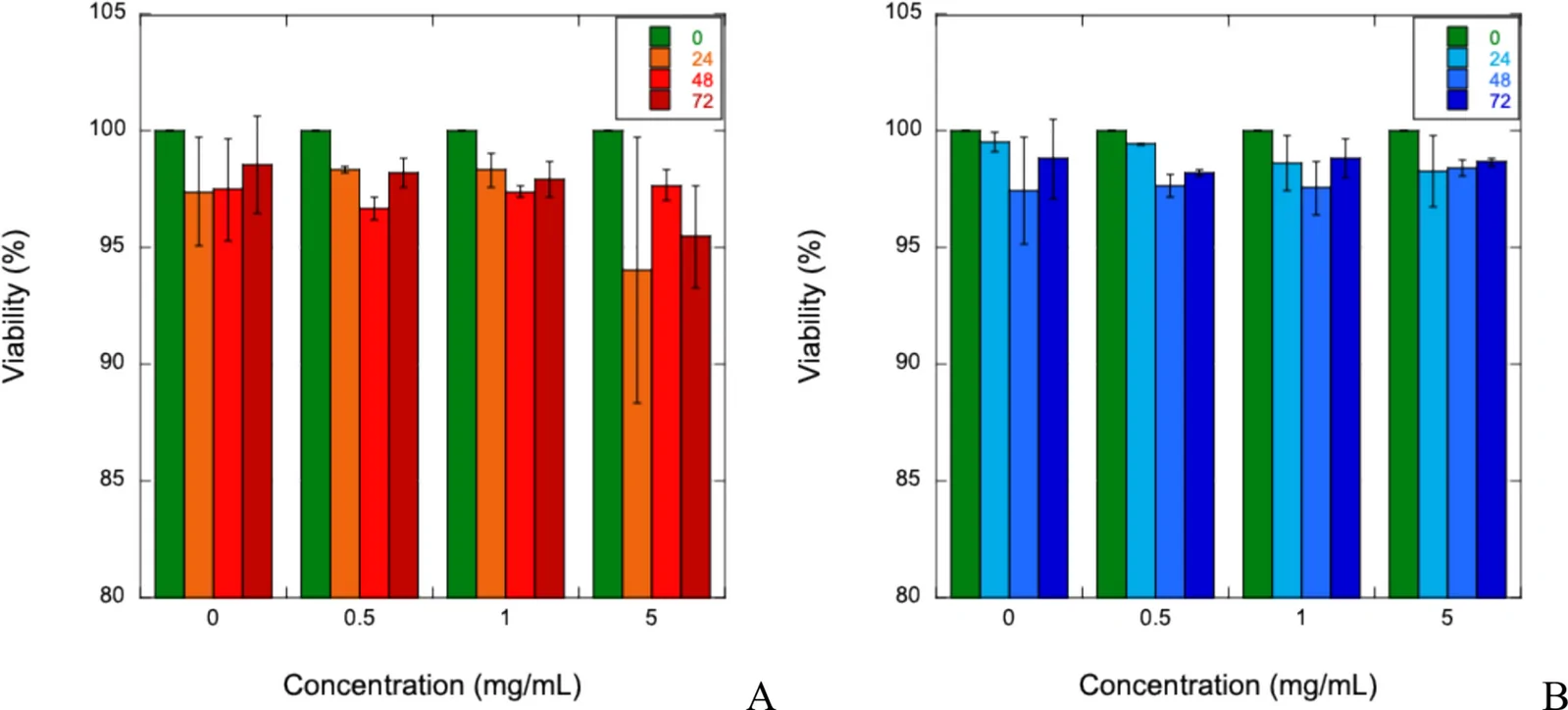
Viability of A549 in the presence of S5 (**A**) and S10 (**B**) at different concentrations: 0.5 mg/mL, 1 mg/mL 5 mg/mL at 4 different time points 0, 24, 48 and 72 h) measured by DAPI labeling. The bars represent the standard deviation (*n* = 3)

To better understand the long term exposure effects, the proliferation rate of A549 cells exposed to different formulation concentrations (namely 0.5, 1 and 5 mg/mL) was performed throught cells counting by flow cytometry after the labelling of death cells. Ther number of cells was close to that of the control for the first 48 h; thereafter, the cells treated with the formulation underwent a reduction of the proliferation rate compared to the control (Fig. S6 in supplementary materials).Then, cell viability was evaluated at all time points by flow cytometry (Fig. 9).

Figure 9 shows a viability close to 100% at all the experimental points for both formulations, confirming their absence of toxicity even at relatively long-term, evidencing a difference from the preliminary viability data obtained with the MTT assay (Fig. 8).

We speculate that the difference between the two assays can be ascribed to the metabolic nature of the MTT test; cells reduce their metabolism but are still alive after 48 h. Indeed, the significant decrease in the viability observed by MTT mirrors the reduction of the proliferation observed after 48 h, suggesting a modulation in the cellular metabolism induced by the treatment.

#### Cellular uptake

The ability of the formulation to be internalized in cells was evaluated by measuring the amount of LT4 in the intracellular compartment after 24 h of exposure to S5 and S10 nano-formulations, compared to the LT4 solution.

**Fig. 10:**
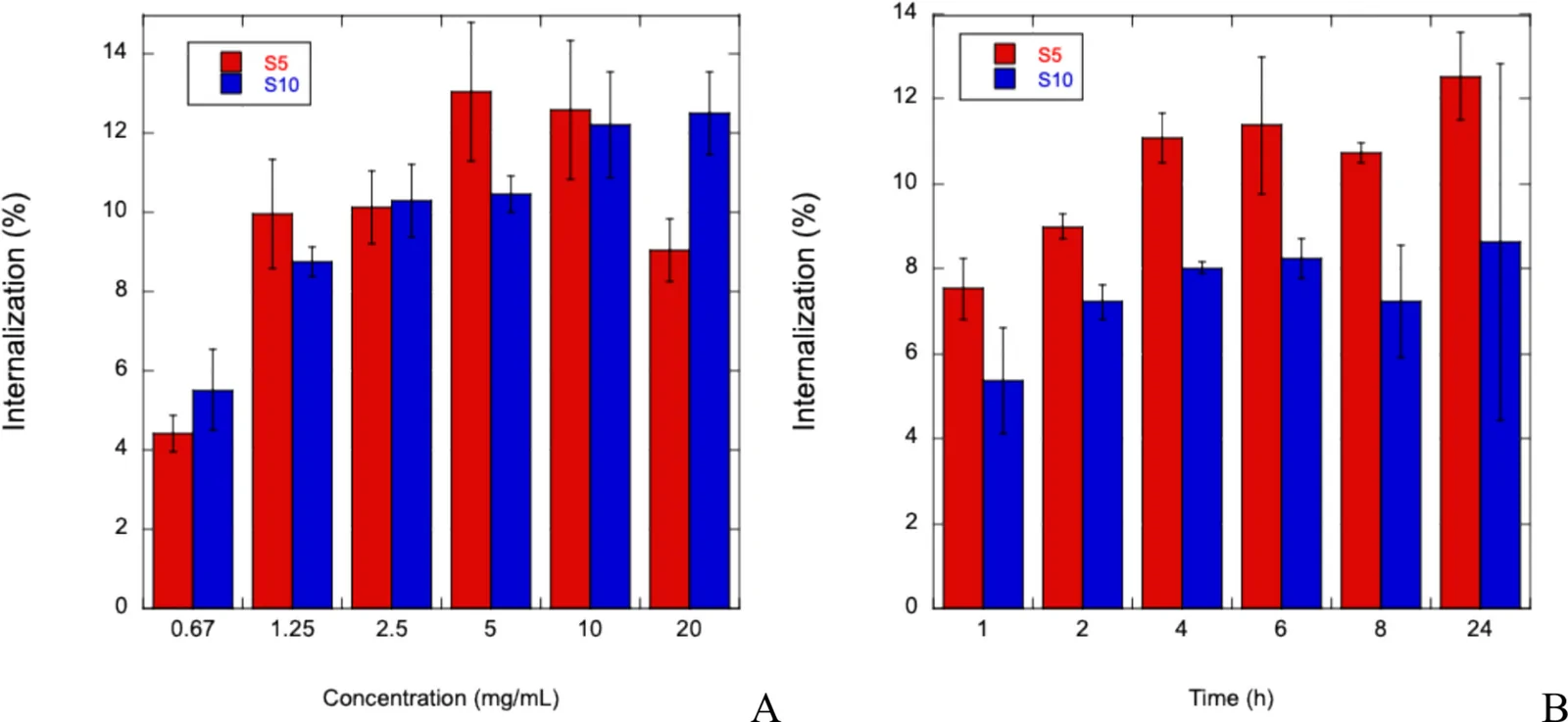
Internalization of LT4 on A549 cells as a function of the formulation concentration after 24 h exposition (**A**), and with respect to the time of exposure with 10 mg/mL of formulations (**B**). The black bars represent the standard deviation (*n* = 4)

LT4 intracellular recovery after the exposition is reported in Fig. 10 as a function of the formulation concentration (Panel A) and of the exposure time (Panel B).

The obtained data evidenced no significant differences in the intracellular recovery between the two formulations at the same concentration, except for the 20 mg/mL, in which the S10 recovery was significantly higher (*p* < 0.05). Interestingly, the process is characterized by an increase in cellular uptake up to about 10% for the concentration of 2.5 mg/mL of the whole formulation (corresponding to 84.5 µg/mL of LT4); thereafter, the percentage of uptake levelled off without significant differences until 20 mg/mL, where a significant reduction (*p* < 0.05) is observed in S5-treated cells.

It is worth underscoring that when the internalization was investigated using an LT4 solution at the maximum concentration achievable (15 µg/mL)^1^ a value of 2.2 ± 0.4% (about 0.3 µg/mL) was obtained, which is statistically different (*p* < 0.01) with respect to the one obtained at the first point in Fig. 10 panel A (0.67 mg/mL of nanosuspension, corresponding to 23 µg/mL of LT4) for both the formulation: 4.43 ± 0.44% (about 1.02 ± 0.08 µg/mL), and 5.51 ± 1.02% (about 1.26 ± 0.2 µg/mL) respectively for S5 and S10.

In addition, the time needed for the uptake was explored using 10 mg/mL of formulations at different time points (1 h, 2 h, 4 h, 6 h, 8 h, and 24 h) (Fig. 10 panel B). The obtained data show how both the formulations are characterized by an increase of the intracellular recovery in the first 4 h, followed by a plateau. However, comparing the two formulations, S5 exhibits a significantly higher intracellular recovery (*p* < 0.05) at all tested time points, with the only exception of the 24 h treatment, in agreement with the data presented in Fig. 10 panel A.

These data demonstrate an enhanced potential for delivering high doses of LT4 using the prepared nanocarrier system compared to a conventional solution. Indeed, formulations S5 or S10, tested at 10 mg/mL for 24 h, afforded an internalizion about 100-fold higher with respect to that obtained with the LT4 almost satrated solution.

The absence of difference observed with the use of high doses coupled to the particle size data (< 200 nm) suggests that the cellular internalization may occur via a receptor-mediated endocytosis mechanism, such as clathrin-coated or caveolae-mediated endocytosis [35], or via phagocytosis [36].

Furthermore, the differences in the intracellular recovery of the two formulations before 24 h of treatment suggest a possible effect of the PVA molecular weight in the "release" of the LT4 nanoparticle, supporting the above-described "matrix effect" (see comment of Fig. S3).

#### ROS production modulation

Due to its role in fibrosis development [37], the cellular oxidative stress was studied by treating the cells under the same conditions used for the proliferation assay.

**Fig. 11:**
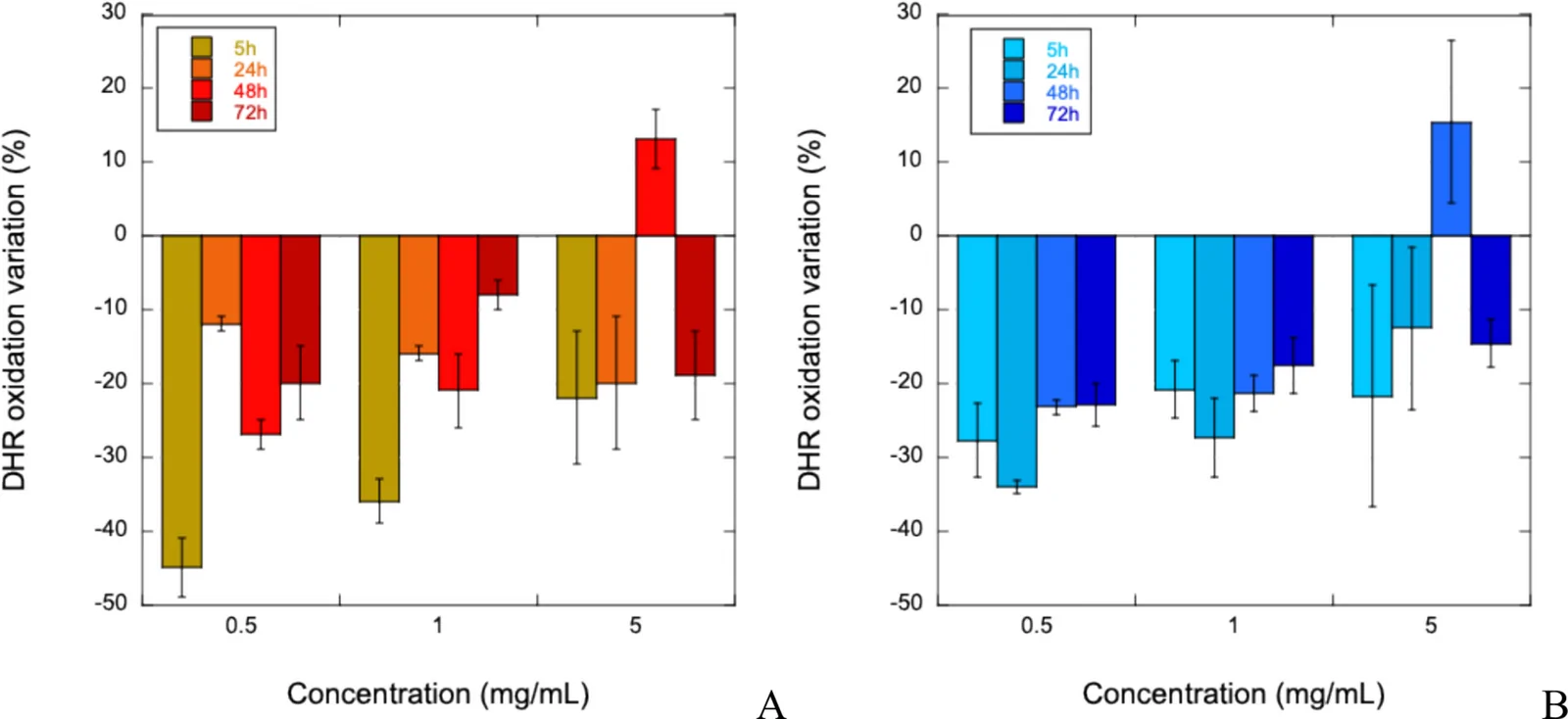
DHR oxidation signal variation after the treatment with S5 (**A**) and S10 (**B**) in three different concentrations (5,1, 0.5 mg/mL) at different exposure times: 5 h, 24 h, 48 h, and 72 h. The bar represents the standard deviation between the sample (*n* = 4)

Figure 11 shows the variation of the DHR oxidation in relative fluorescence as the ratio between the signal recorded with the treated cells and that of the non-treated ones.

It could be appreciated how the DHR fluorescence is systematically reduced after the treatment with S5 at all the tested concentrations and at all the time points (panel A). The only exception was represented by the treatment with the highest concentration (5 mg/mL) after 48 h: in this case, the signal increased by about 12%. The higher reduction (nearly 45%) was obtained by treating with 0.5 mg/mL of formulation for 5 h. For the other times points, the treatment led to a reduction in the DHR oxidation ranging between 10 and 30%.

A similar trend was presented by formulation S10 (Fig. 11, panel B): DHR fluorescence is systematically reduced at all concentrations and at all time points. In this case, the higher reduction (about 33%) was obtained with 0.5 mg/mL of formulation for 5 h. Again, at the other time points, the formulation reduced the DHR oxidation between 10 and 30%. Curiously, as observed for S5, the only exception to this trend was represented by the high concentration after 48 h: in this case, the signal increased by about 15%. This peculiar effect remains to be clarified and will be further investigated in the future.

Overall, these data support the hypothesis of metabolic modulation by the administered nanoformulated LT4, wich show a statistically significant effect in the reduction of oxydative species produced by the cells.

### Mitochondrial functionality: evaluation of the mitochondrial membrane potential (MMP)

The last metabolic aspect explored was the integrity and functionality of the mitochondria after exposure to the formulation in the same conditions used for the proliferation test.

**Fig. 12:**
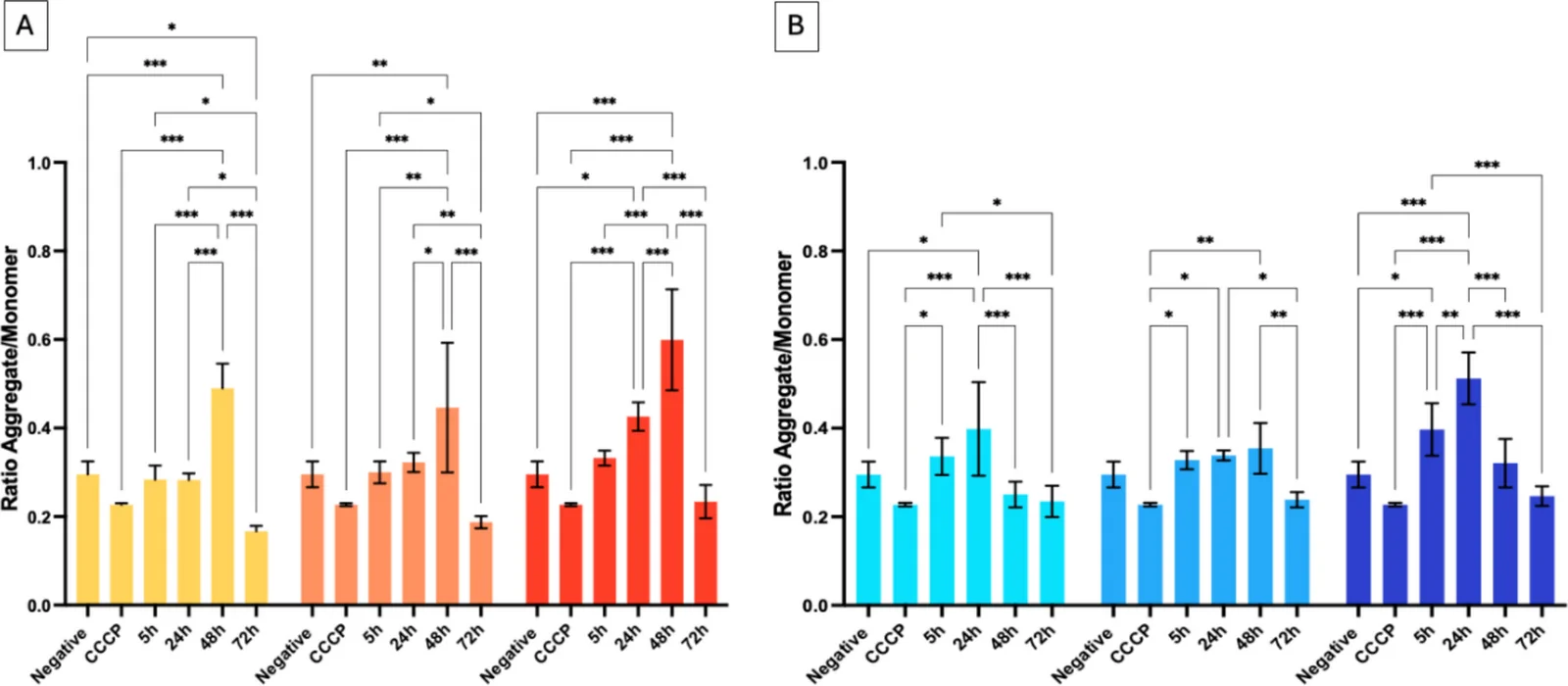
Red/Green channels intensity ratio (Aggregate/Monomer) of A549 treated with S5 (**A**) or S10 (**B**) at the three different concentrations (0.5,1,5 mg/mL from left to right) compared to the positive control (CCCP) The bar represents the standard deviation (*n* = 4). (*P < 0.033, **P < 0.002, *** *P* < 0.001)

Figure 12 shows that Formulation S5 (panel A) at the lower concentration (0.5 mg/mL) did not determine a significant change with respect the negative control for the first 24 h; after 48 h, a significant (*P* < 0.001) increase in the MMP (compared to the untreated) was observed. However, after 72 h, the MMP was significantly lower than the negative control and not significantly different from the positive one. Treatment with 1 mg/mL afforded a similar behavior. A further dose increase (5 mg/mL) gave rise to a similar trend, although at higher values of the red/green ratio.

Data relevant to S10 formulation are reported in panel B. The trend was similar to that observed with Formulation S5 with the only difference represented by the shift of the higher aggregate-monomer ratio at 24 h rather than 48h. Anyhow, the value recorded at 48 h was not statistically different from the negative control.

It can be inferred that both formulations can modulate the MMP and are non-toxic for the first 48 h of exposure, whereas the mitochondrial polarization decreases after 72 h in all samples likely due to the long term exposure and/or the increase in the mitophagy process. It is also worth underscoring that the tumoral origin of the used cell line may influence the observed variability and output values.

## Conclusions

Idiopathic pulmonary fibrosis is an unmet medical need requiring urgent, clinically relevant solutions. The repositioning of already marketed drugs represents an excellent opportunity for the rapid development of new medicinal products, provided that a suitable drug delivery system allows overcoming technological and biopharmaceutical barriers.

The present work introduces a new strategy for producing an innovative drug delivery system for lung administration capable of delivering LT4 in relatively high doses to reverse the fibrotic state in IPF patients. This outcome is achieved through a nanomedicinal formulation capable of efficiently reaching the deep lung.

One of the key elements is the capability of the formulation to generate a stable colloidal system, upon contact with biological fluids, with an LT4 concentration much higher than that obtainable with the net drug solution, and able to be internalized by lung epithelial cells without significantly affecting cell viability and proliferation.

This work provides preliminary data on the ability of the formulations to modulate the cell metabolic activity by reducing the oxidative stress and increasing the mitochondrial activity.

Finally, we demonstrate the feasibility of a powder for pulmonary administration of LT4 able to reach the intracellular target effectively and efficiently, and provide, a first element for paving the way towards the clinical translation and development of a medicinal product capable of offering a valid therapeutic response to IPF patients.

## Supplementary Information

Below is the link to the electronic supplementary material.

Supplementary file1 (DOCX 775 KB)

## Data Availability

The datasets generated during and/or analysed during the current study are available from the corresponding author on reasonable request.

## Footnotes

1 Complete medium with 0.05% v/v DMSO.
