# Supplementary material for: Engineered levothyroxine dry powder for inhalation to treat idiopathic pulmonary fibrosis
Source: Drug Deliv Transl Res. 2026 Feb 7;16(10):3728–45. doi: 10.1007/s13346-026-02064-2 (PMC13619685; doi:10.1007/s13346-026-02064-2)
Supplement: Supplementary file 1 [file 13346_2026_2064_MOESM1_ESM.docx]

**Supplementary materials of “Engineered levothyroxine dry powder for inhalation to treat idiopathic pulmonary fibrosis”.**

**G.Bazzoli et al.**

**Table S1** Laser and emission channel used on the cytometer MacsQuant 10

| **Laser** | **Filter (nm)** | **Fluorophore** |
| --- | --- | --- |
| Violet (405 nm) | 450/50 | DAPI |
| Blue (488 nm) | 525/50 | DHR  JC-1 monomeric form |
|  | 585/40 | JC-1 Aggregate form |

**Fig. S1** Saturated LT4 solution in water (left) and 1% w/v PVA aqueous solution (right) after filtration with a 0.45 µm syringe filter.

***Fig S2*** *Equivalent projected area diameter of the sub-micron particle determined by ImageJ analysis; S5 formulation (red) S10 formulation (blue).*

**Fig S3** S5 (Panel A) and S10 (Panel B) size distribution of formulations with different concentrations 10 mg/mL (green), 2 mg/mL (black), 1 mg/mL (blue), 0.5 mg/mL (red).

**Table S2** MaDLS measurement of S5 at different concentrations, DS represents the standard deviation (n = 3)

| *S5*  *(mg/mL)* | *Peak 1* | *SD* | *% intensity* | *DS* | *Peak 2* | *SD* | *% intensity* | *DS* |
| --- | --- | --- | --- | --- | --- | --- | --- | --- |
| 10 | 466 | 3.25 | 86.43 | 1.645 | 149.6 | 5.742 | 12.63 | 0.746 |
| 2 | 399.5 | 58.99 | 62.03 | 12.15 | 162.9 | 12.15 | 36.31 | 9.597 |
| 1 | 285.3 | 97.08 | 67.65 | 5.009 | 196.3 | 81.85 | 31.22 | 33.956 |
| 0.5 | 226 | 37.5 | 81.47 | 19.01 | 421.7 | 13.19 | 27.21 | 13.6 |

**Table S3** MaDLS measurement of S10 at different concentrations, DS represents the standard deviation (n = 3)

| *S10 (mg/mL)* | *Peak 1* | *SD* | *% intensity* | *DS* | *Peak 2* | *SD* | *% intensity* | *DS* |
| --- | --- | --- | --- | --- | --- | --- | --- | --- |
| *10* | 438.6 | 25.6 | 87.71 | 7.388 | 144.1 | 14.46 | 8.377 | 2.626 |
| *2* | 377.8 | 69.56 | 67.56 | 4.811 | 161.1 | 10.22 | 30.58 | 5.705 |
| *1* | 329.7 | 83.9 | 86.38 | 23.59 | 182.1 | - | 40.87 | - |
| *0.5* | 264.2 | 36.76 | 66.19 | 19.75 | 262.6 | 123.7 | 33.81 | 19.75 |

**Table S4** Particle size distributions of S5 and S10 formulation after resuspension (0.5 mg/mL) in ultrapure water measured by NTA at time zero and after 24 months of storage at room temperature.

| *Time (months)* | *Formulation* | *DV_10_ (nm)* | *DV_50_ (nm)* | *DV_90_ (nm)* |
| --- | --- | --- | --- | --- |
| *0* | *S5* | *57.6* ± 3.91 | *175.6* ± 10.11 | *399.6* ± 16.22 |
|  | *S10* | *83.6* ± 10.06 | *198.0* ± 10.17 | *431.2* ± 18.93 |
| *24* | *S5* | *97* ± 2.65 | *169* ± 5.50 | *328* ± 17.89 |
|  | *S10* | *93.4* ± 3.91 | *160* ± 8.86 | *276.2* ± 17.62 |

**Table S5** Aerodynamic parameters of S10 formulation after 24 months of storage at room temperature.

| *Emitted dose (%)* | 102.27 ± 3.52 |
| --- | --- |
| *MMAD (µm)* | 4.50 ± 0.97 |
| *GSD (µm)* | 4.07 ± 1.33 |
| *Fine particle fraction (%) <5 µm* | 70.45 ± 2.62 |
| *Extra-fine particle fraction (%) <2 µm* | 60.55 ±1.38 |

**Fig. S4** A549 cell metabolic activity (%, MTT assay) after 4h exposition to LT4 solution at different concentrations, the bars represent the standard deviation (n=5).

Figure S5: A549 cell viability after 4h exposition to PVA at two molecular weights. In red PVA 13-23 kDa, in blue PVA 31-50 kDa; the bars represent the standard deviation (n=5).

Figure S6: Proliferation of A549 in the presence of S5 (panel A) and S10 (panel B) at different concentrations: 5 mg/mL (red), 1 mg/mL (blue),0.5 mg/mL (black), untreated control (green), the bars represent the standard deviation (n=3).
